# Upright MDCT evaluation of gravity-induced bronchial collapse and paradoxical lung volume reduction in severe thoracic lordoscoliosis: A case report

**DOI:** 10.1016/j.radcr.2026.07.113

**Published:** 2026-08-25

**Authors:** Akira Inoue, Kota Watanabe, Satoshi Suzuki, Yoshitake Yamada, Masaya Nakamura, Masahiro Jinzaki

**Affiliations:** a Department of Orthopedic Surgery, Keio University School of Medicine, Tokyo, Japan; b Department of Radiology, Keio University School of Medicine, Tokyo, Japan

**Keywords:** Upright MDCT, Respiratory function, Thoracic lordoscoliosis, Posterior spinal fusion

## Abstract

A 21-year-old male with severe thoracic lordoscoliosis presented with progressive dyspnea, which worsened in the upright position. Because standing whole-spine radiographs showed postural exacerbation of lordoscoliosis, upright multidetector computed tomography (MDCT) was performed in addition to conventional supine imaging to assess dynamic anatomical changes under gravitational influence. Preoperative upright MDCT revealed significant dynamic bronchial collapse. The right main bronchus diameter decreased from 3.23 mm in the supine position to 1.15 mm in the upright position, representing a 64.4% reduction. Simultaneously, lung volume paradoxically decreased by 30% upon standing. These findings were consistent with posture-dependent worsening of deformity-induced airway compromise and intrathoracic volume reduction under gravitational loading. Postoperatively, upright lung volume increased by 77.5%, with resolved bronchial compression. Upright MDCT may provide valuable adjunctive diagnostic support by quantifying deformity-induced respiratory dysfunction that may not be fully captured by conventional supine imaging, particularly when symptoms are posture-dependent.

## Introduction

Untreated excessive thoracic lordoscoliosis can lead to life-threatening pulmonary dysfunctions [1,2]. Surgical intervention is often necessitated to prevent further respiratory deterioration, although consensus is lacking on whether correction surgery consistently improves pre-existing pulmonary impairment [1,3,4].

A significant diagnostic challenge is that conventional supine CT tests may not capture the dynamic pathophysiology occurring in the upright position. Traditional imaging can underestimate airway compression and lung volume reduction exacerbated by gravitational load. Recently developed upright multi-detector CT (MDCT) allows for the quantitative evaluation of anatomical structures in a standing position [5]. We present a case where upright MDCT visualized posture-induced dynamic bronchial collapse and paradoxical lung volume reduction. This advanced imaging modality may be particularly useful when a patient’s symptoms are posture-dependent or when conventional supine imaging does not fully explain the degree of respiratory impairment.

## Case report

**Fig. 1:**
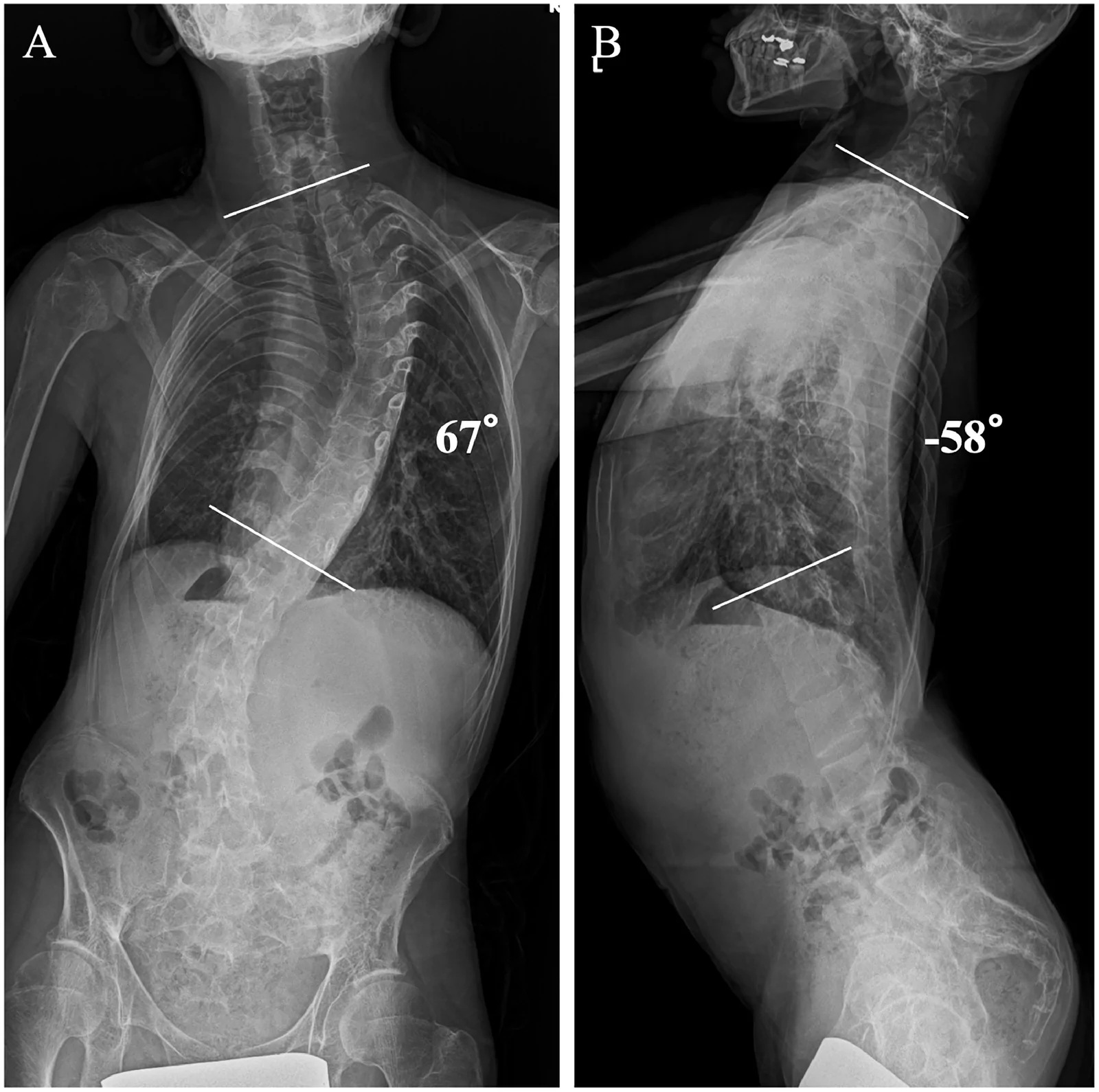
Preoperative upright whole-spine radiographs. (A) Upright anteroposterior radiograph. (B) Upright lateral radiograph. Upright radiographs showed scoliosis of 67 degrees from T1 to T12 (A) and lordosis of −58 degrees from C7 to L1 (B).

A 21-year-old male diagnosed with Noonan syndrome was referred to our department due to progressive dyspnea worsening in the upright position, suspected to be related to lordoscoliosis. Pulmonary function tests revealed both restrictive and obstructive ventilation disorders (Table 1). Preoperative standing whole-spine radiographs showed scoliosis of 67 degrees and lordosis of −58 degrees, reducing to 44 degrees and −41 degrees on supine radiographs (Fig. 1). This discrepancy strongly suggested that the spinal alignment and, consequently, his thoracic capacity were significantly influenced by gravitational load, leading to the decision to perform advanced dynamic imaging.

**Table 1:** Preoperative and postoperative pulmonary function test results.

	Preoperative	Postoperative
VC (L)	1.50 (35.5%)	2.21 (52.1%)
FVC (L)	1.43 (33.9%)	2.13 (50.6%)
FEV1 (L)	0.80 (21.1%)	1.90 (50.9%)
FEV1/FVC	55.9	89.2
PEF (L/s)	2.21 (20.9%)	5.70 (54.9%)

Values in parentheses indicate predicted values.

FEV1, forced expiratory volume in 1 second; FEV1%, FEV1/FVC ratio; FVC, forced vital capacity; PEF, peak expiratory flow; VC, vital capacity.

To quantify the impact of gravity on the respiratory structures, comparative MDCT scans (Aquilion ONE and prototype TSX-401R, Canon Medical Systems) were performed in both supine and upright positions during breath-hold at end-inspiration. Both scans utilized a low-dose protocol: 100 kVp, rotation speed of 0.5 seconds, and a pitch factor of 0.813, with 0.5-mm slice thickness reconstructed using AIDR 3D. Lung volumes were automatically measured using a CAD-integrated workstation (Synapse Vincent, Fujifilm), which performed precise lung extraction. The minimum luminal diameter of the right main bronchus was measured at the site of maximum compression in both postures.

**Fig. 2:**
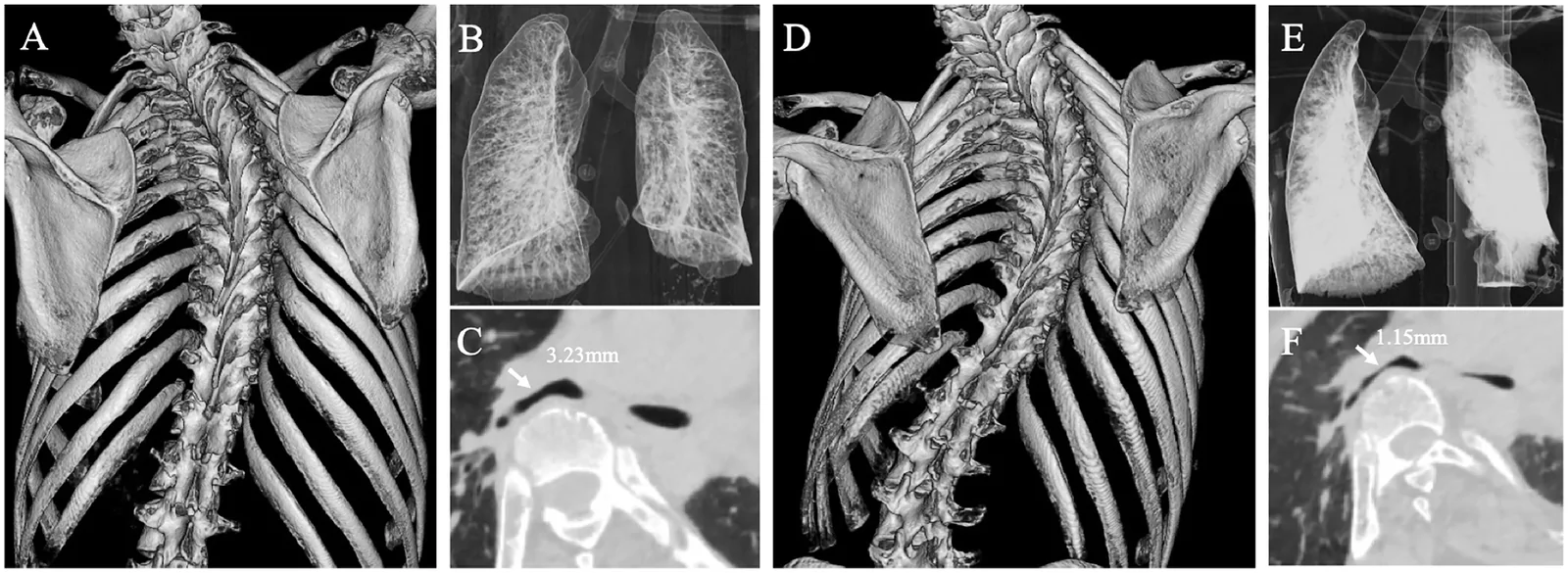
Preoperative multidetector computed tomography (MDCT) in supine and upright positions. (A) Three-dimensional reconstructed image of the thoracic cavity in the supine position obtained from MDCT. (B) Three-dimensional reconstructed image of the lung volume in the supine position obtained from MDCT. (C) Axial lung-window image of the right main bronchus in the supine position obtained from MDCT. (D) Three-dimensional reconstructed image of the thoracic cavity in the upright position obtained from MDCT. (E) Three-dimensional reconstructed image of the lung volume in the upright position obtained from MDCT. (F) Axial lung-window image of the right main bronchus in the upright position obtained from MDCT. Lung volumes measured on MDCT were 2541 mL in the supine (B) and 1794 mL in the upright position (D), indicating a 30% decrease upon standing. The right main bronchus was further compressed in the upright position (C: supine; F: upright), with the minimum luminal diameter decreasing from 3.23 to 1.15 mm (64.4% reduction).

Conventional supine MDCT already demonstrated compression of the right main bronchus by the lordotic vertebrae, with a minimum luminal diameter of 3.23 mm. The diagnostic evaluation was further refined by upright MDCT, which revealed a more pronounced exacerbation of the pathophysiology under physiological loading. Upon standing, the protruding vertebrae further encroached upon the airway, reducing the bronchial diameter to 1.15 mm. Furthermore, volumetric analysis showed that the patient’s lung capacity paradoxically decreased by 30% (from 2541 mL supine to 1794 mL upright), a notable departure from the physiological increase typically observed in healthy individuals (Fig. 2).

These quantitative observations provided valuable adjunctive diagnostic support for surgical planning by demonstrating posture-dependent worsening of bronchial compression and concomitant lung volume reduction under gravitational loading. Although the indication for surgery was based on the overall clinical and radiographic assessment, upright MDCT strengthened the rationale for posterior spinal fusion and provided an objective baseline for postoperative comparison.

**Fig. 3:**
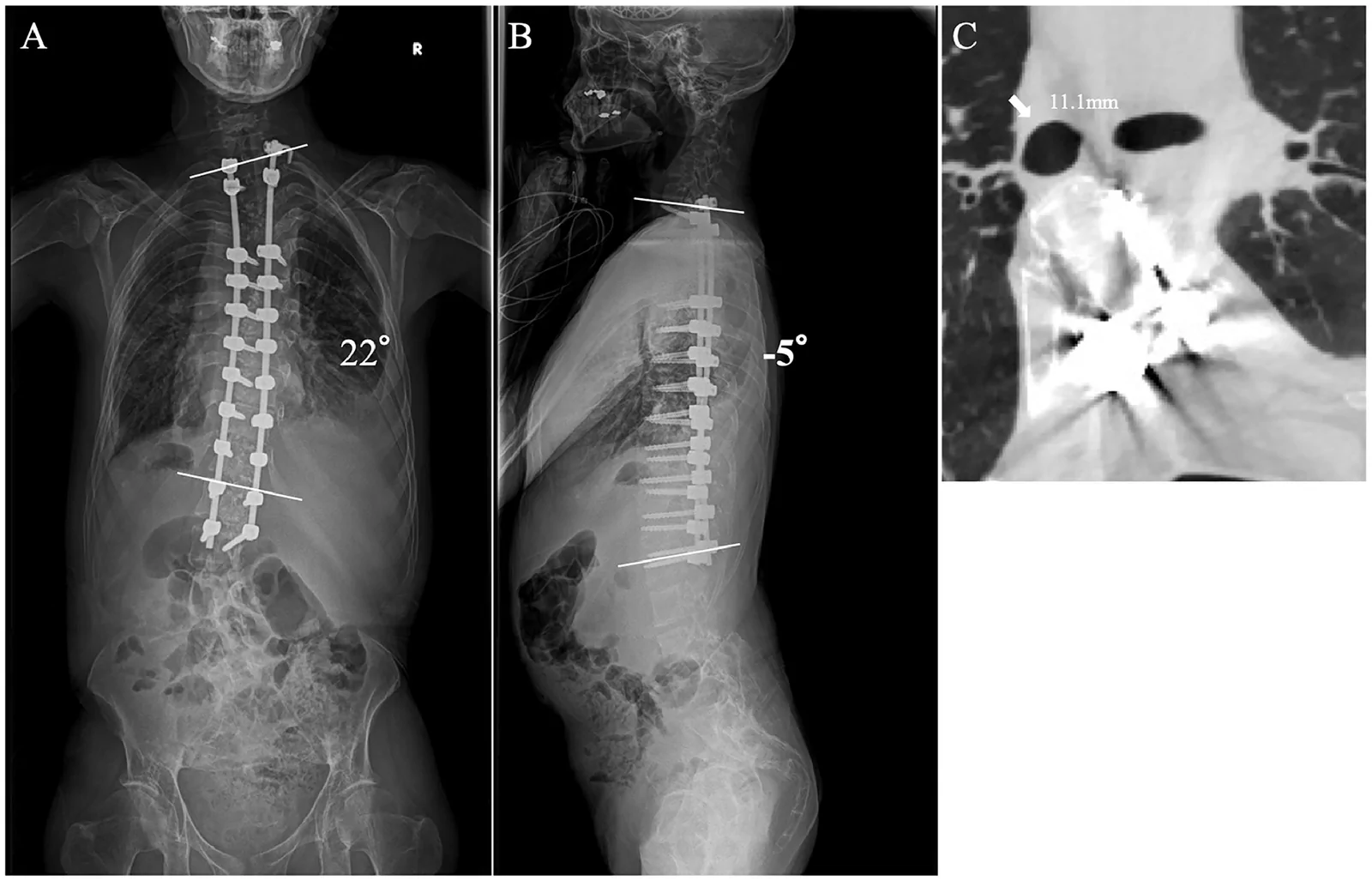
Postoperative upright whole-spine radiographs and upright MDCT at 4-year follow-up. (A) Postoperative upright anteroposterior radiograph. (B) Postoperative upright lateral radiograph. (C) Axial lung-window image of the right main bronchus in the upright position obtained from postoperative MDCT. Both the scoliosis and lordosis were corrected to 22 degrees from T1 to T12 (A) and −5 degrees from C7 to L1 (B). Postoperative upright MDCT showed increased lung volume of 3185 mL; a 77.5% increase compared to the preoperative level; and decompression of the right main bronchus (11.1 mm) (C).

The patient underwent posterior spinal correction and fusion from T1 to L1. The surgery successfully corrected the lordoscoliosis (postoperative Cobb angle: 22°, lordosis: −5°), immediately alleviating the respiratory compromise. At the 4-year follow-up, postoperative upright MDCT confirmed complete resolution of the bronchial compression and a 77.5% increase in upright lung volume (3185 mL) compared to the preoperative level (Fig. 3). Although moderate respiratory impairment persisted, these findings were consistent with postoperative clinical improvement (Table 1).

## Discussion

To the best of our knowledge, this is the first report to visualize and quantify the pathophysiology of deformity-induced respiratory impairment in the upright position using CT imaging. While previous studies have primarily attributed respiratory impairment in severe lordoscoliosis to bronchial compression by lordotic vertebrae and reduced thoracic compliance, our findings provide a novel perspective on the dynamic nature of these changes under gravitational load [6,7].

The key physiological finding in this case was the paradoxical reduction in lung volume upon standing. In healthy individuals, lung volume typically increases by 9%-10% in the upright position due to the caudal shift of the diaphragm and the alleviation of abdominal pressure [8]. In contrast, this patient exhibited a 30% decrease in lung capacity when transitioning from a supine to an upright posture. This inverse pattern was consistent with posture-dependent worsening of the thoracic deformity and associated encroachment on the intrathoracic space. Notably, upright MDCT demonstrated greater bronchial narrowing under physiological loading than conventional supine MDCT, revealing a dynamic 64.4% reduction in the right main bronchus diameter (from 3.23 mm supine to 1.15 mm upright). This posture-induced dynamic bronchial collapse provided valuable adjunctive diagnostic support for posterior spinal fusion.

At the 4-year follow-up, decompression of the right main bronchus and increased upright lung volume provided objective postoperative imaging findings consistent with clinical improvement. While further cases are needed to clarify the broader relationship between thoracic lordoscoliosis and dynamic respiratory function, upright MDCT served as a valuable diagnostic adjunct for surgical decision-making in this particular case.

## Conclusion

We propose that upright MDCT may provide valuable adjunctive diagnostic support for assessing posture-dependent respiratory impairment in selected patients with complex spinal deformity, particularly when conventional supine imaging may underestimate symptom-relevant airway compression and lung volume reduction.

## Patient consent

This case report was covered by institutional broad consent obtained at admission. Additionally, the patient provided written informed consent for publication of the case and accompanying images.
